# Supplementary material for: Synthesis, Pharmacokinetic Profile, Anticancer Activity and Toxicity of the New Amides of Betulonic Acid—In Silico and In Vitro Study
Source: Int J Mol Sci. 2024 Apr 20;25(8):4517. doi: 10.3390/ijms25084517 (PMC11050400; doi:10.3390/ijms25084517)
Supplement: Supplementary file 1 [file ijms-25-04517-s001.zip › ijms-2918594-supplementary.pdf]

Supplementary materials

Synthesis, pharmacokinetic profile, anticancer activity and toxicity of the new amides of betulonic acid – in silico and in vitro study

Bębenek Ewa[1], Rzepka Zuzanna[2], Hermanowicz Justyna[3,4], Chrobak Elwira[1], Surażyński Arkadiusz[5], Beberok Artur[2], Wrześniok Dorota[2,*]

[1] Department of Organic Chemistry, Faculty of Pharmaceutical Sciences in Sosnowiec, Medical University of Silesia in Katowice, 4 Jagiellońska Str., 41-200 Sosnowiec, Poland
[2] Department of Pharmaceutical Chemistry, Faculty of Pharmaceutical Sciences in Sosnowiec, Medical University of Silesia in Katowice, 4 Jagiellońska Str., 41-200 Sosnowiec, Poland
[3] Department of Pharmacodynamics, Medical University of Bialystok, Mickiewicza 2c, 15-222 Bialystok, Poland
[4] Department of Clinical Pharmacy, Medical University of Bialystok, Mickiewicza 2c, 15-222 Bialystok, Poland
[5] Department of Medicinal Chemistry, Medical University of Bialystok, Kilinskiego 1, 15-089 Bialystok, Poland

*Corresponding author.
E-mail address: dwrzesniok@sum.edu.pl

LIST OF CONTENTS

Experimental

A. Synthesis - Materials and methods

Figure S1. ^{1}H NMR, compound EB170

Figure S2. ^{13}C NMR, compound EB170

Figure S3. IR, compound EB170

Figure S4. EI MS, compound EB170

Figure S5. HRMS, compound EB170

Figure S6. ^{1}H NMR, compound EB171

Figure S7. ^{13}C NMR, compound EB171

Figure S8. IR, compound EB171

Figure S9. EI MS, compound EB171

Figure S10. HRMS, compound EB171

Figure S11. ^{1}H NMR, compound EB173

Figure S12. ^{13}C NMR, compound EB173

Figure S13. IR, compound EB173

Figure S14. EI MS, compound EB173

Figure S15. HRMS, compound EB173

B. Lipophilicity

Table S1. The experimental (R_{M0} and $logP_{TLC}$) and literature ($logP_{lit}$) lipophilicity values for reference compounds (mobile phase acetone:buffer Tris)

Table S2. The theoretical values of lipophilicity for betulonic acid and its amide derivatives

Table S3. The correlation matrix for theoretically obtained lipophilicity parameters of tested compounds

Table S4. The correlation between the molecular descriptors and the R_{M0} values for betulonic acid and its amide derivatives

Table S5. The correlation between the anticancer activity (IC_{50}) and the R_{M0} values for betulonic acid and its derivatives

A. *Synthesis - Materials and methods*

The chemical reagents were purchased from the Sigma-Aldrich. All melting points (mp) were determined using an Electrothermal IA 9300 apparatus, and are uncorrected. ^{1}H and ^{13}C NMR experiments were performed on a Bruker Avance III 600 spectrometer in $CDCl_3$, as solvent. Chemical shifts (δ) were referenced to the signal of the $CDCl_3$ and are given in parts per million (ppm). The determined coupling constants (*J*) are reported in Hertz (Hz). Infrared spectra were recorded on an IRAffinity-1 Shimadzu spectrometer. EI MS spectra were recorded on a Finnigan MAT 95 instrument. HRMS spectra were taken on a Bruker Impact II instrument mass spectrometer. The course of the reaction was monitored using Silica gel 60 254F plates (Merck). The spots of the chromatograms were visualized by spraying with an 10% ethanolic solution of H_2SO_4 and heating to 100 °C. Purification was conducted by column chromatography using silica gel 60 (0.063-0.200 mm).

Figure S1. ^{1}H NMR, compound EB170

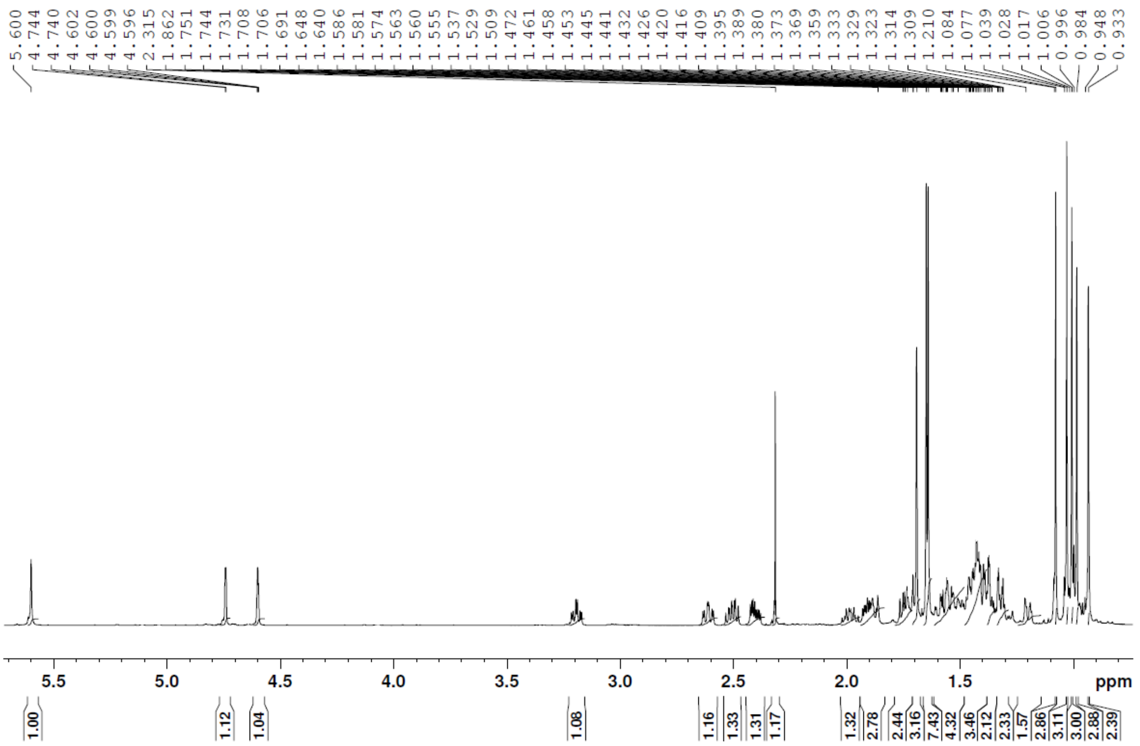

Figure S2. ^{13}C NMR, compound EB170

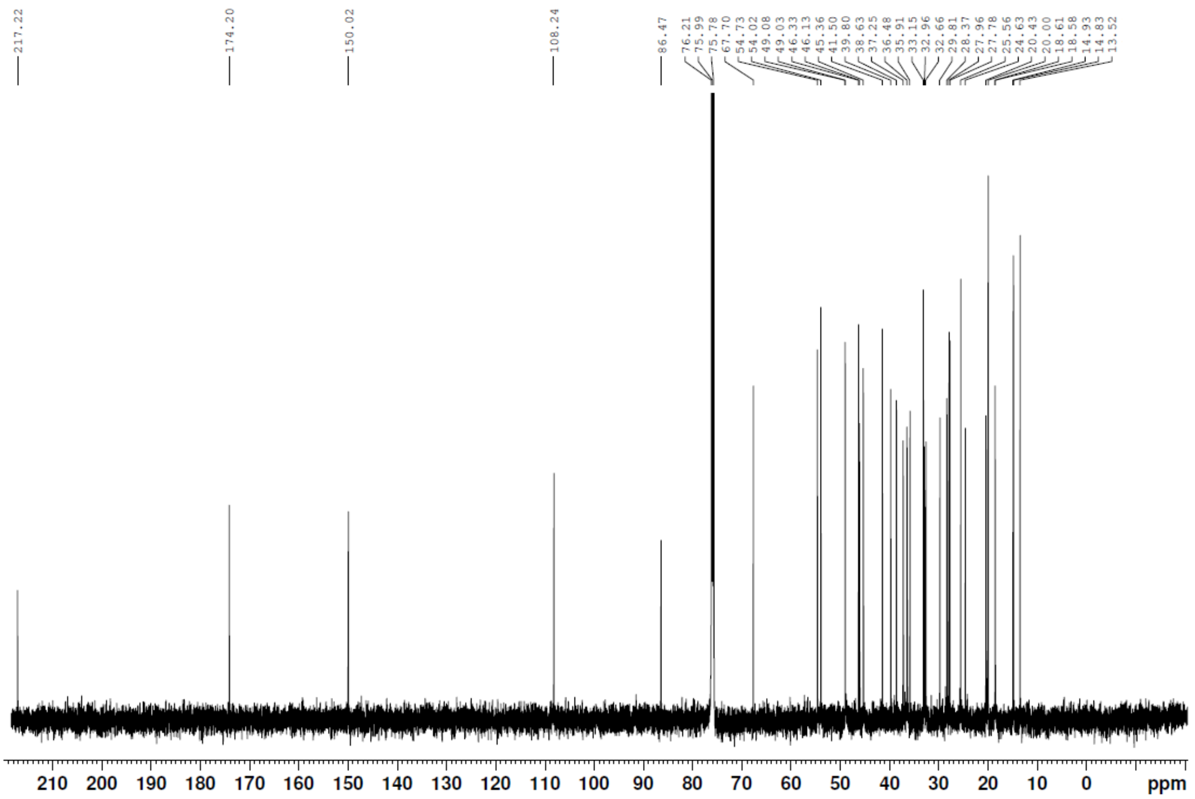

Figure S3. IR, compound EB170

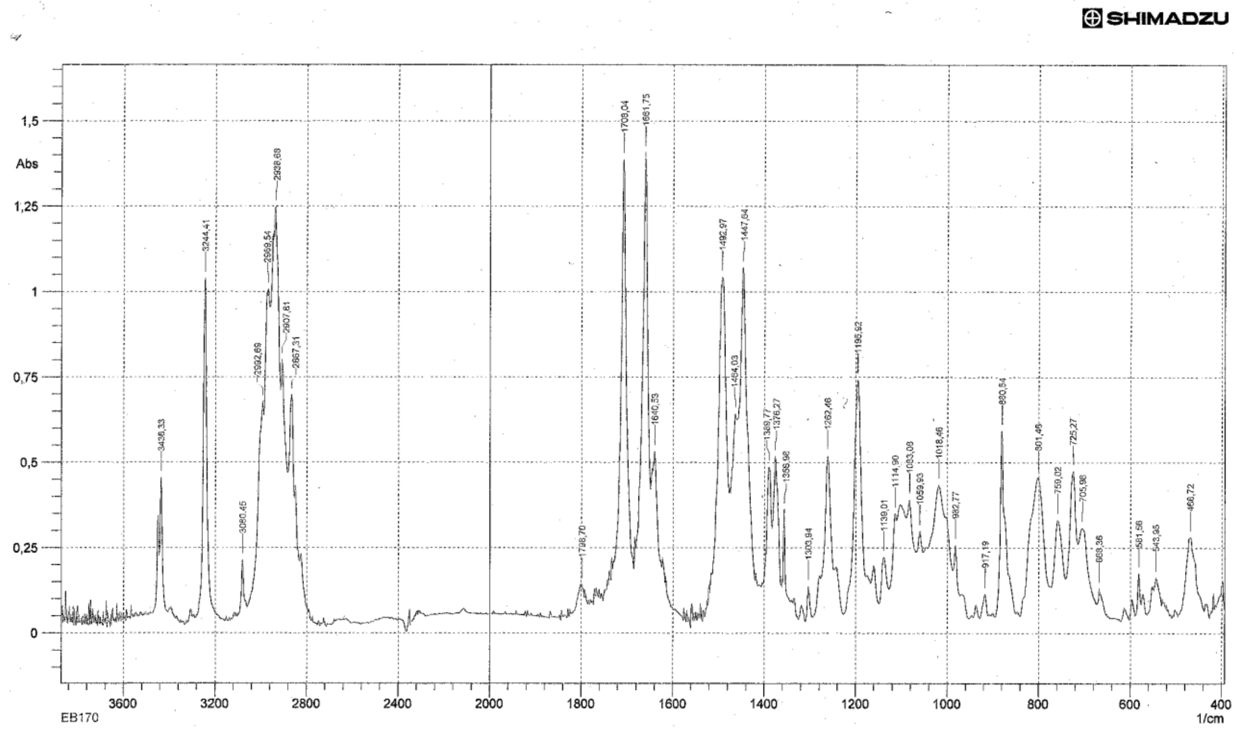

Figure S4. EI MS, compound EB170

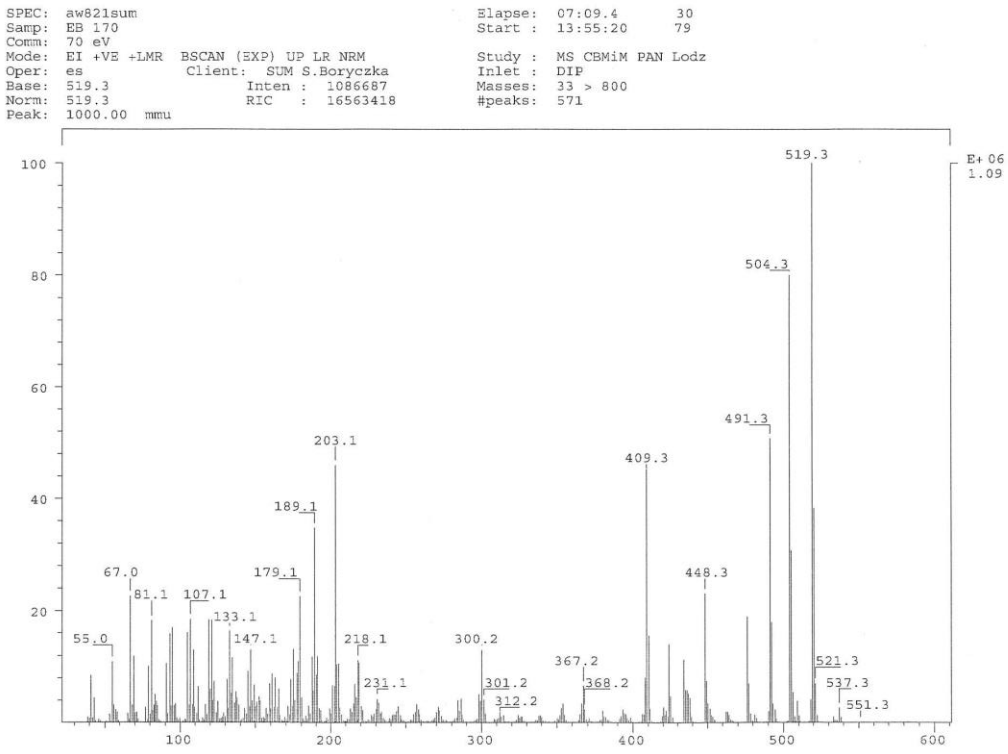

Figure S5. HRMS, compound EB170

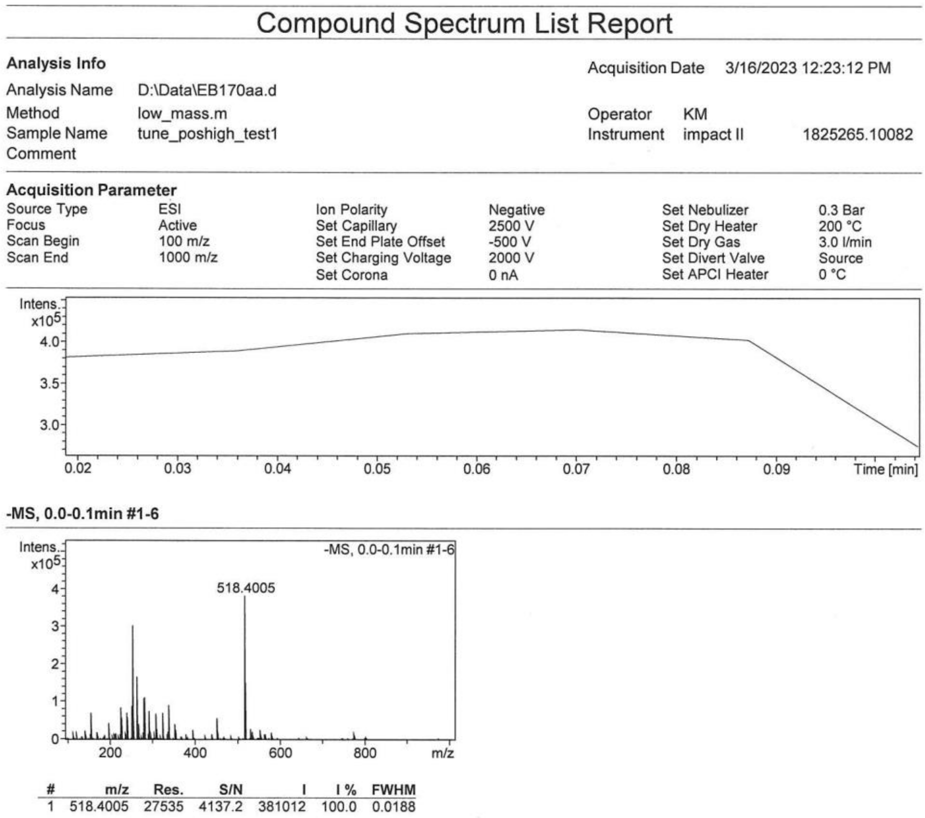

Compound Spectrum List Report

Analysis Info

Analysis Name	D:\Data\EB170aa.d	Acquisition Date	3/16/2023 12:23:12 PM
Method	low_mass.m	Operator	KM
Sample Name	tune_poshigh_test1	Instrument	impact II 1825265.10082
Comment			

Acquisition Parameter

Source Type	ESI	Ion Polarity	Negative	Set Nebulizer	0.3 Bar
Focus	Active	Set Capillary	2500 V	Set Dry Heater	200 °C
Scan Begin	100 m/z	Set End Plate Offset	-500 V	Set Dry Gas	3.0 l/min
Scan End	1000 m/z	Set Charging Voltage	2000 V	Set Divert Valve	Source
		Set Corona	0 nA	Set APCI Heater	0 °C

-MS, 0.0-0.1min #1-6

#	m/z	Res.	S/N	I	I %	FWHM
1	518.4005	27535	4137.2	381012	100.0	0.0188

Figure S6. ^{1}H NMR, compound EB171

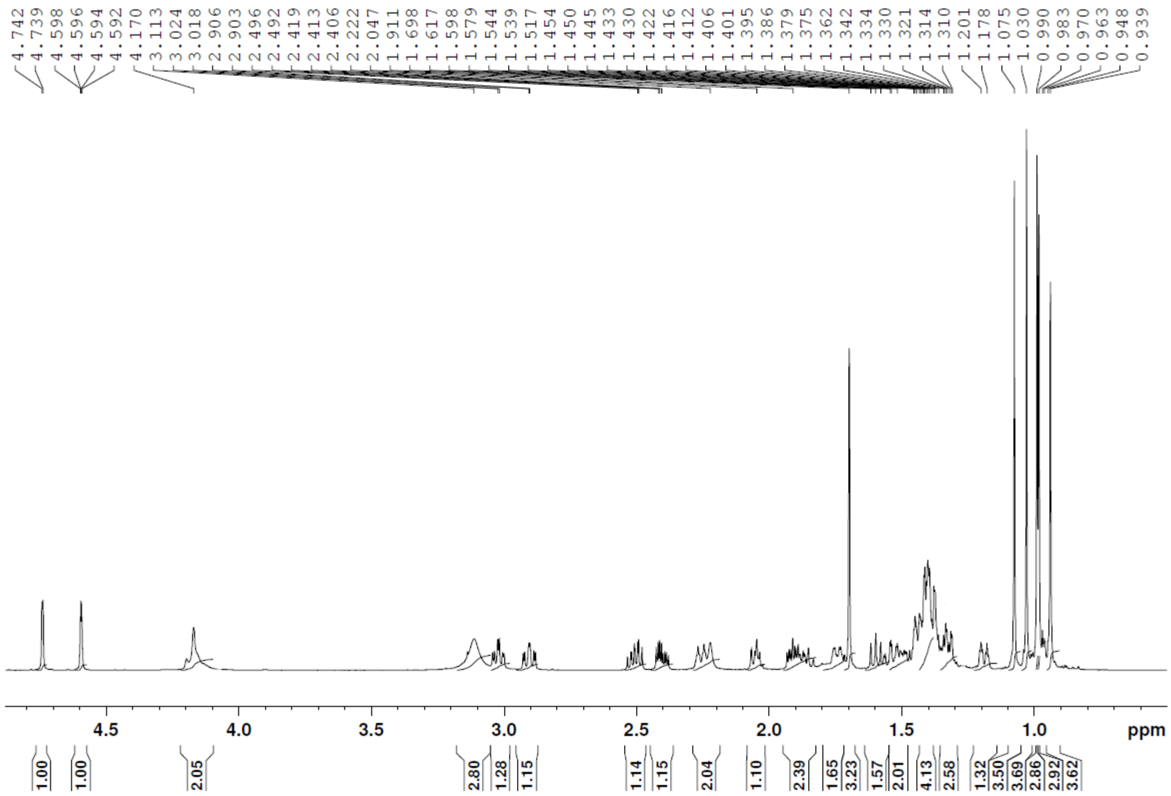

Figure S7. ^{13}C NMR, compound EB171

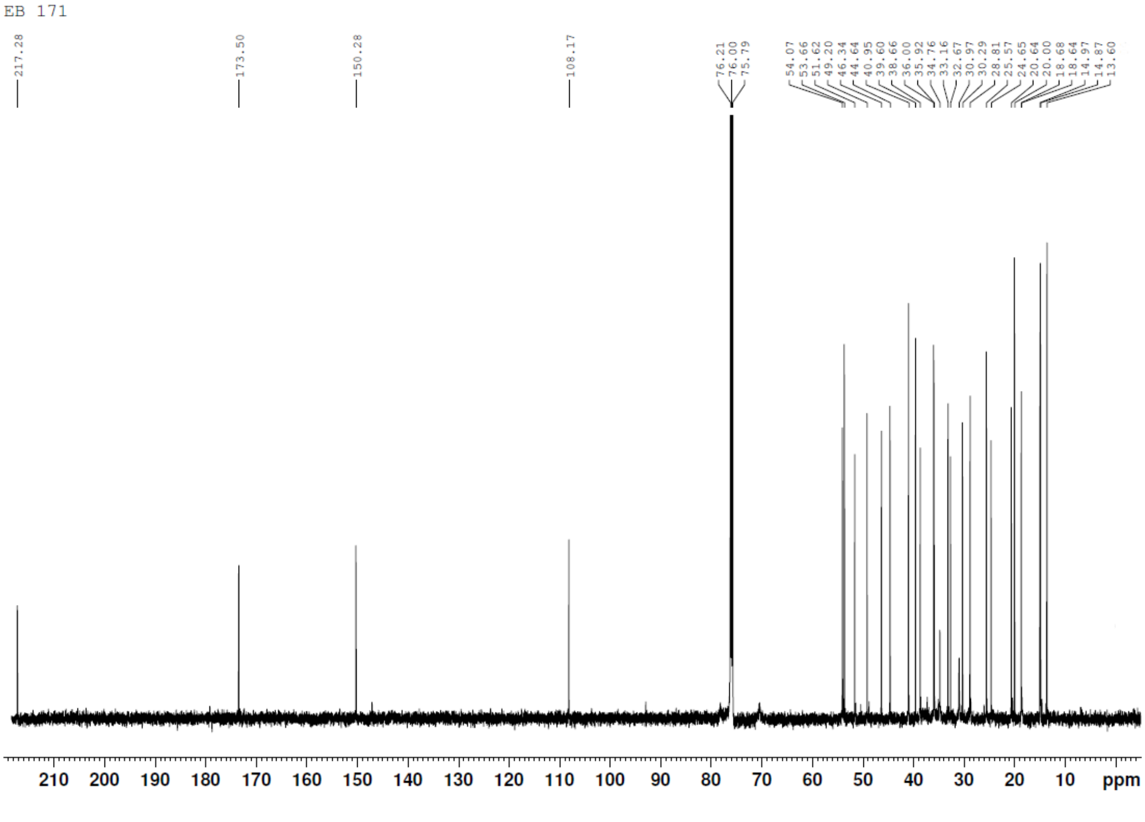

Figure S8. IR, compound EB171

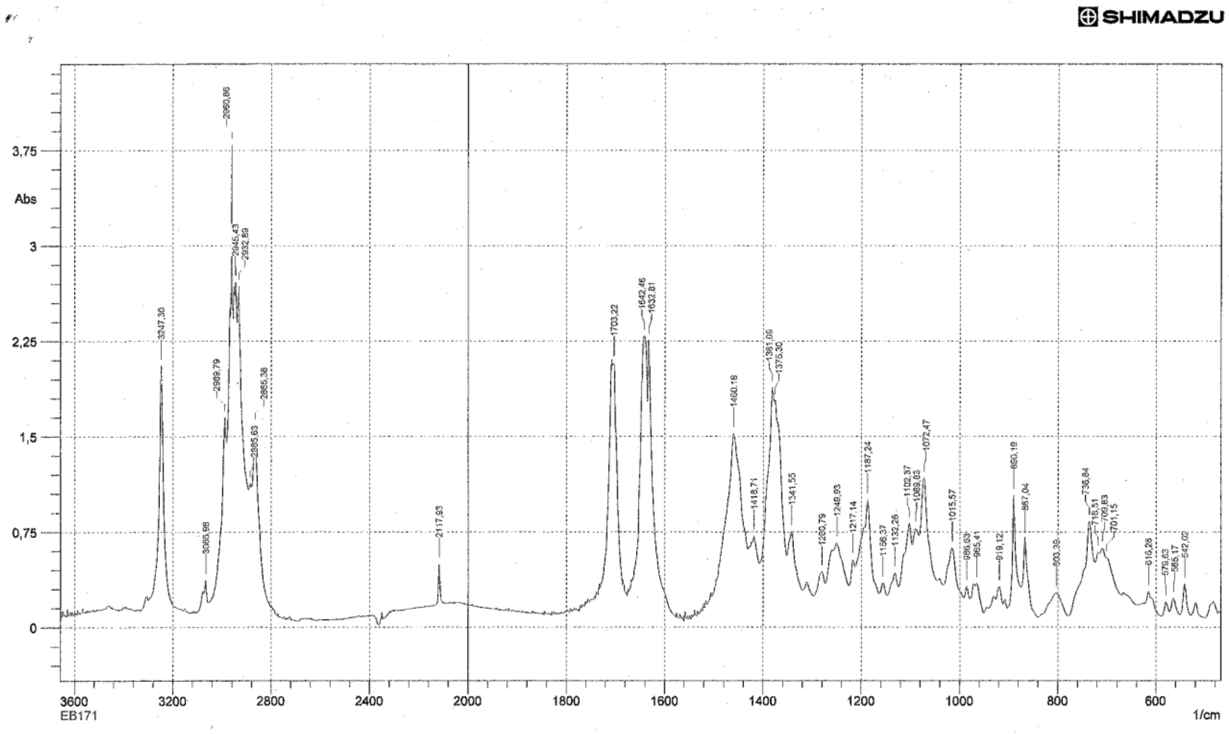

Figure S9. EI MS, compound EB171

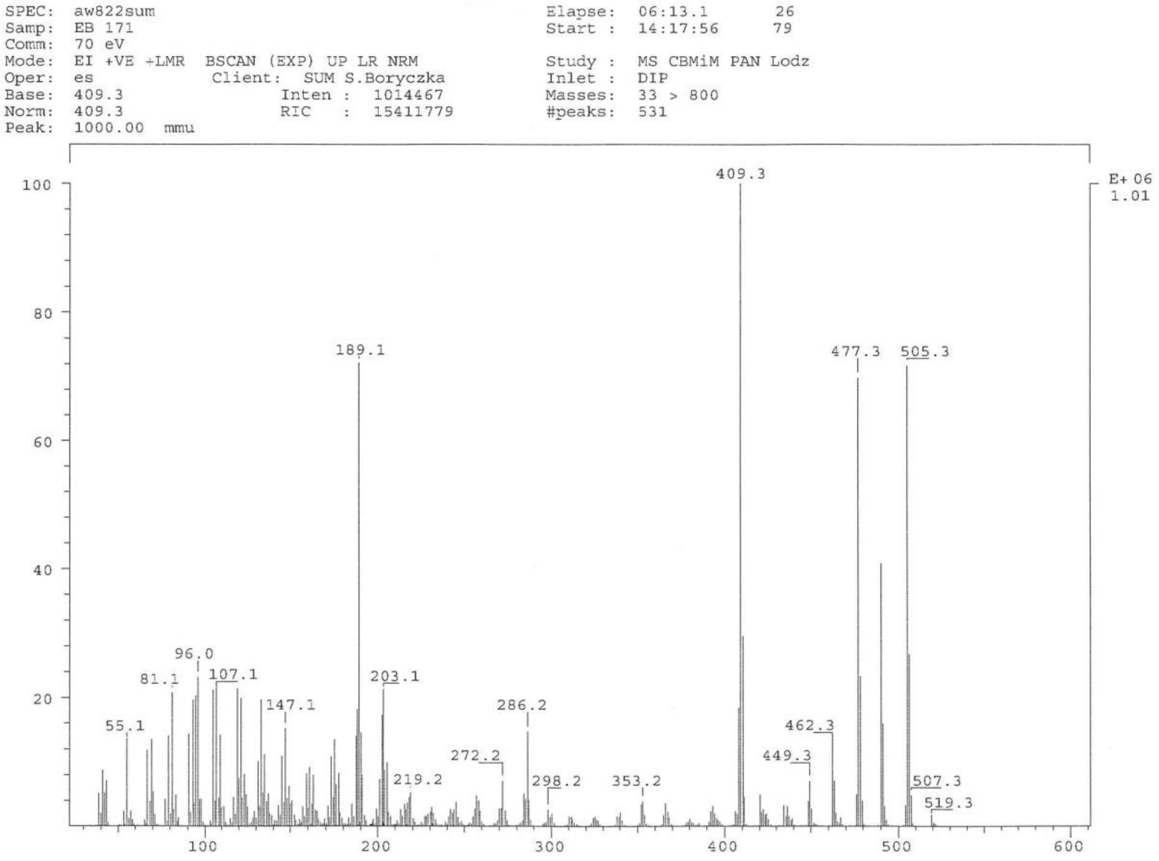

Figure S10. HRMS, compound EB171

Compound Spectrum List Report

Analysis Info

Analysis Name	D:\Data\EB171 APCI.d	Acquisition Date	3/17/2023 2:22:24 PM
Method	APCI_low_ mass_negative.m	Operator	KM
Sample Name	tune_poshigh_test1	Instrument	impact II 1825265.10082
Comment			

Acquisition Parameter

Source Type	APCI	Ion Polarity	Negative	Set Nebulizer	2.0 Bar
Focus	Active	Set Capillary	4000 V	Set Dry Heater	200 °C
Scan Begin	100 m/z	Set End Plate Offset	-500 V	Set Dry Gas	4.0 l/min
Scan End	1000 m/z	Set Charging Voltage	2000 V	Set Divert Valve	Source
		Set Corona	15000 nA	Set APCI Heater	450 °C

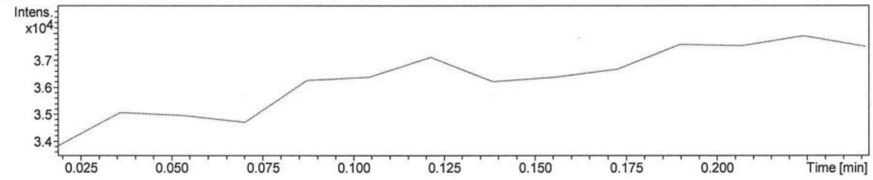

-MS, 0.0-0.2min #1-14

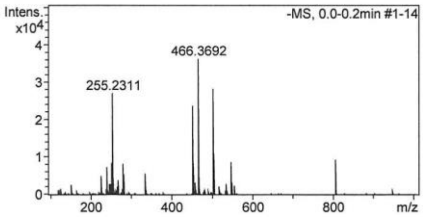

#	m/z	Res.	S/N	I	I %	FWHM
1	255.2311	19345	867.7	27071	74.5	0.0132
2	453.3374	21724	822.9	23631	65.0	0.0209
3	466.3692	22361	1290.5	36331	100.0	0.0209
4	504.3850	22898	1062.3	27866	76.7	0.0220

Figure S11. ^{1}H NMR, compound EB173

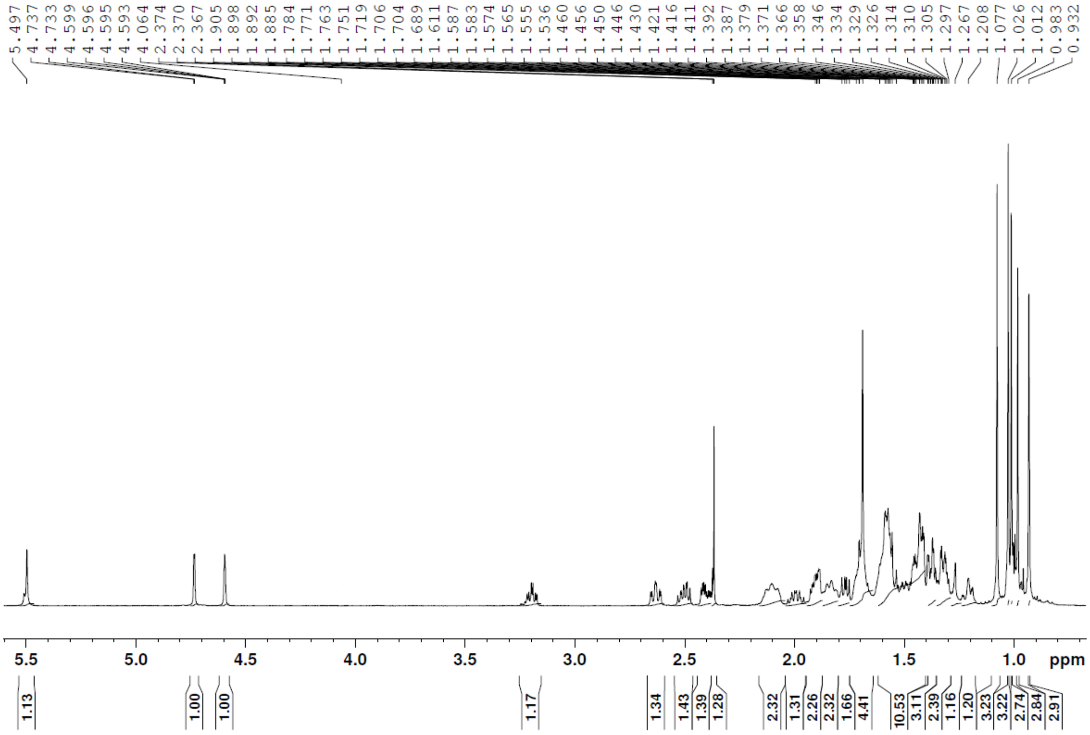

Figure S12. ^{13}C NMR, compound EB173

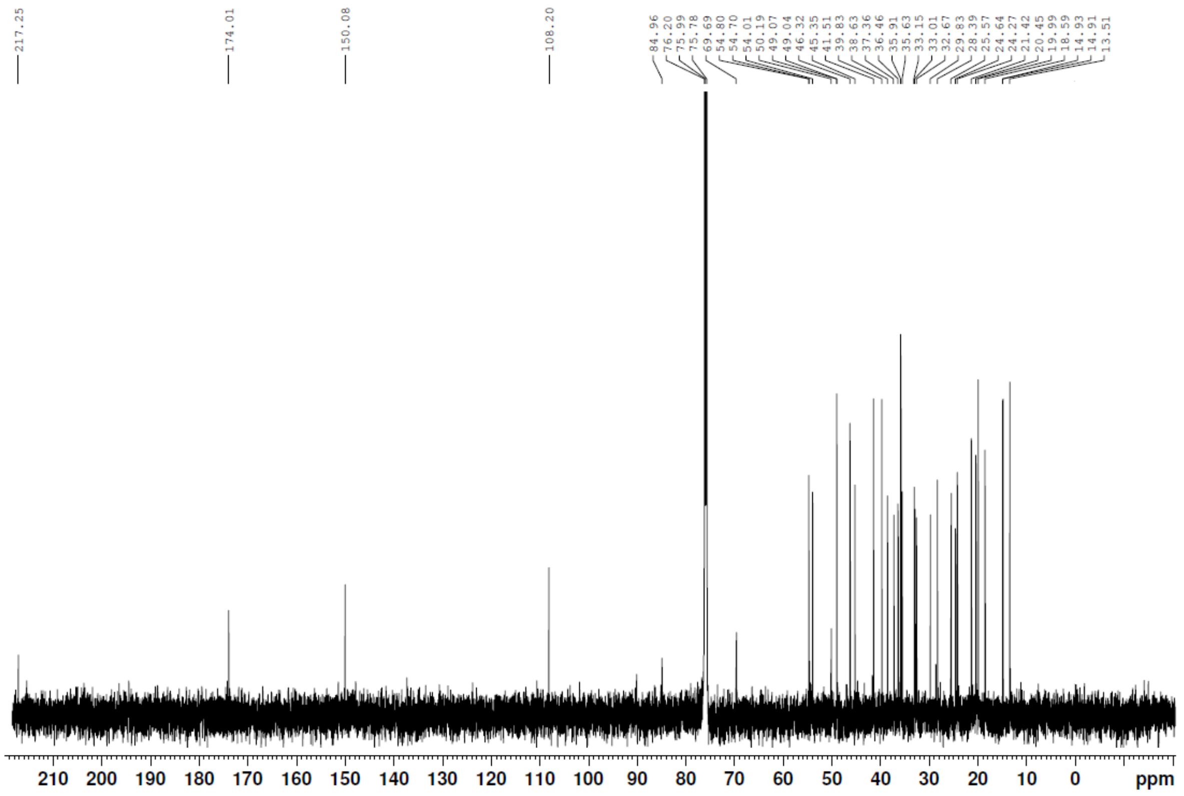

Figure S13. IR, compound EB173

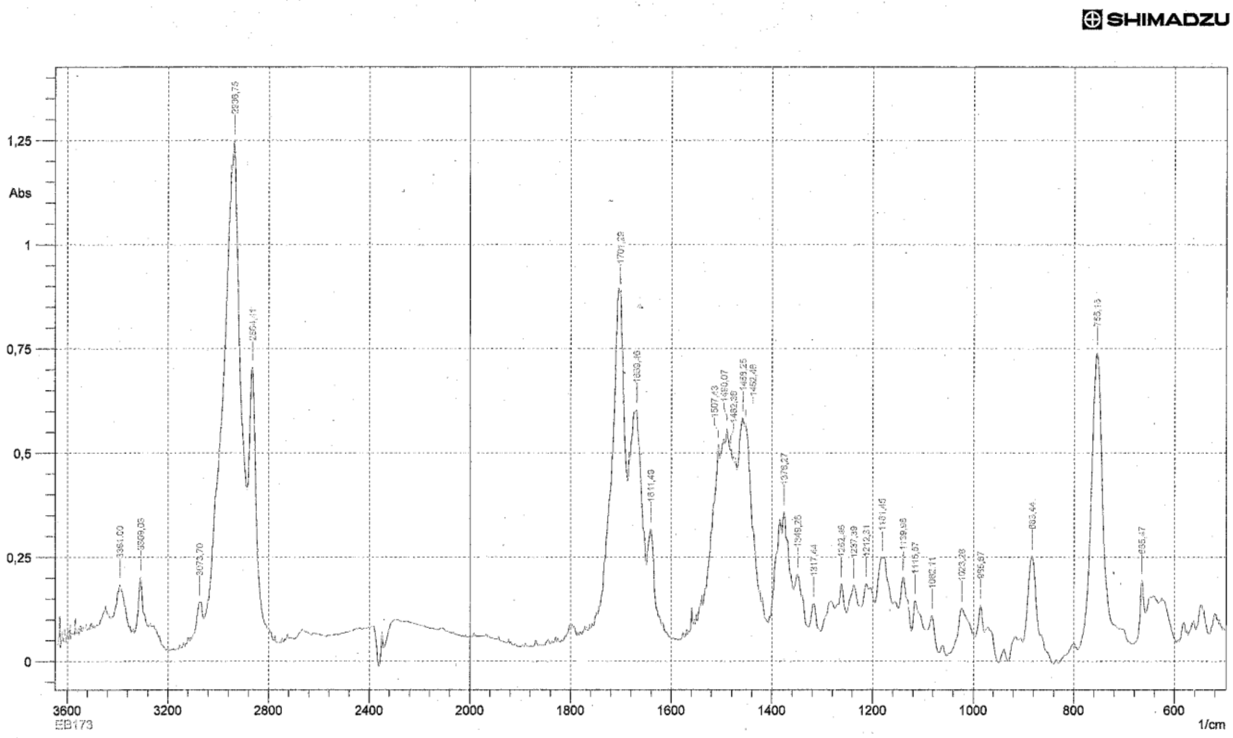

Figure S14. EI MS, compound EB173

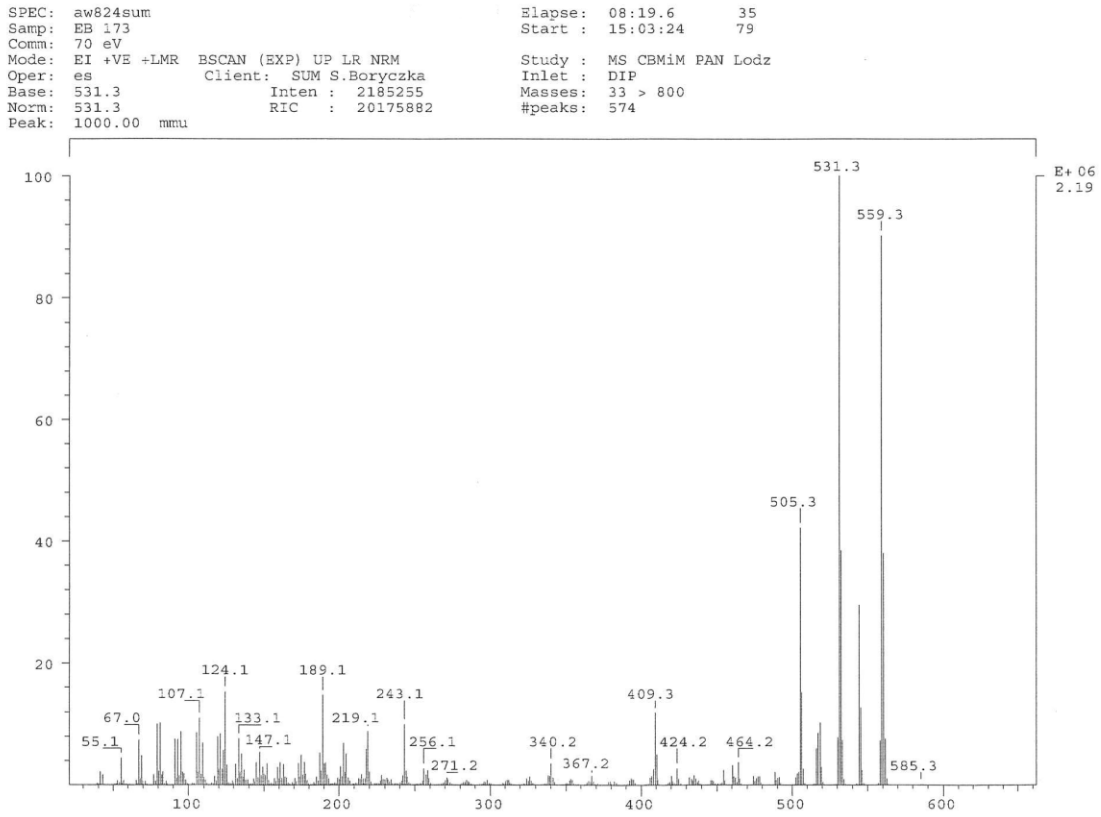

Figure S15. HRMS, compound EB173

Compound Spectrum List Report

Analysis Info

Acquisition Date 3/16/2023 12:28:00 PM

Analysis Name D:\Data\EB173.d
Method low_mass.m
Sample Name tune_poshigh_test1
Comment

Operator KM
Instrument impact II 1825265.10082

Acquisition Parameter

Source Type	ESI	Ion Polarity	Negative	Set Nebulizer	0.3 Bar		
Focus	Active	Set Capillary	2500 V	Set Dry Heater	200 °C		
Scan Begin	100 m/z	Set End Plate Offset	-500 V	Set Dry Gas	3.0 l/min		
Scan End	1000 m/z	Set Charging Voltage	2000 V	Set Divert Valve	Source		
		Set Corona	0 nA	Set APCI Heater	0 °C		

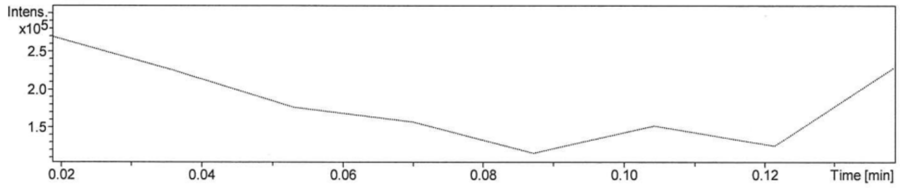

-MS, 0.0-0.1min #1-8

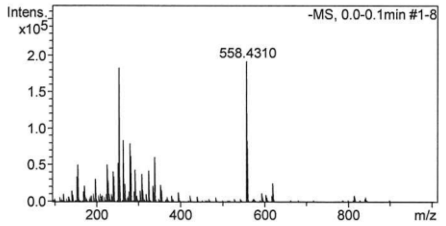

#	m/z	Res.	S/N	I	I %	FWHM
1	558.4310	26005	2909.4	192500	100.0	0.0215

B. *Lipophilicity*

Chromatographic determination of lipophilicity parameters.

The experimental determination of the lipophilicity of the tested substances was carried out using reversed-phase thin-layer chromatography (RP-TLC). For this purpose, aluminum plates covered with modified silica gel RP-18 with F254s fluorescent indicator were used, purchased from Merck. Samples for testing were prepared by dissolving 2 mg of the appropriate substance (standards listed in table S1 or newly synthesized amide derivatives) in 2 ml of chloroform. 2 μL of the obtained solutions was taken and placed on chromatographic plates, which were then developed in chromatographic chambers saturated with eluent vapors. Mixtures of acetone and Tris buffer (aqueous solution of tris-hydroxymethyl)aminomethane in concentration 0.2 M, pH 7.4) were used as mobile phases in volume proportions taking into account the acetone content from 60 to 90% (every 5%). The chromatographic determination was performed in triplicate for each tested substance. The chromatograms were sprayed with a solution of concentrated sulfuric acid and ethanol (in a volume ratio of 1:10), and then the plates were heated to a temperature of 110°C until spots of the analyzed substances were visible. For all substances, in each eluent system, the distances from the starting line to the center of the spot (a) and from the starting line to the eluent front line were measured, and on this basis the retardation factor ($R_{f)}$ was calculated (1) and then the value of the R_M coefficient (2):

$$R_f = a/b \qquad (1)$$

$$R_M = \log [(1/R_f)-1] \qquad (2)$$

The dependence of the R_M parameter on the percentage concentration of acetone in the mobile phase (C) is presented by the Soczewiński-Wachtmeister equation:

$$R_M=R_{M0}+bC \qquad (3)$$

where: b – slope of the regression graph.

Extrapolation of the acetone content in the eluent system to zero allows the determination of the chromatographic lipophilicity parameter R_{M0}.

To determine the calibration curve (dependence of logP on R_{M0}), eight reference compounds were used, for which the literature $\log P_{lit}$ values are in the range of 1.21–6.38 [1]. R_{M0} values for these compounds were determined in the same way as for the tested amide derivatives and betulonic acid (Table S1).

[1]. Kadela-Tomanek, M.; Jastrzębska, M.; Marciniec, K.; Chrobak, E.; Bębenek, E.; Boryczka, S. Lipophilicity, pharmacokinetic properties, and molecular docking study on SARS-CoV-2 target for betulin triazole derivatives with attached 1,4-quinone. Pharmaceutics 2021, 13, 781. https://doi.org/ 10.3390/pharmaceutics13060781.

Table S1. The experimental (R_{M0} and $logP_{TLC}$) and literature ($logP_{lit}$) lipophilicity values for reference compounds (mobile phase acetone:buffer Tris)

Reference compound	R_{M0}	$logP_{lit}$	b	*r*	$logP_{TLC}$
acetanilide	0.57	1.21	-0.01	0.982	1.31
prednisone	0.72	1.62	-0.01	0.989	1.48
4-bromoacetophenone	1.82	2.43	-0.02	0.985	2.73
benzophenone	2.23	3.18	-0.03	0.989	3.19
anthracene	3.03	4.45	-0.04	0.993	4.10
dibenzyl	3.51	4.79	-0.04	0.994	4.64
9-phenylanthracene	3.99	6.01	-0.05	0.996	5.19
DDT	5.20	6.38	-0.06	0.997	6.56

b is the slope and *r* is the correlation coefficient for the linear relationship $R_M = R_{M0} + bC$

The following calibration curve equation was used to determine the experimental lipophilicity values ($logP_{TLC}$) of the tested amide derivatives and betulonic acid:

$$\log P_{TLC} = 1.1332\ R_{M0} + 0.6683\ (r = 0.992)$$

Table S2. The theoretical values of lipophilicity for betulonic acid and its amide derivatives

Compound	iLOGP	XLOGP3	WLOGP	MLOGP	SILICOS-IT
**betulonic acid**	3.62	7.89	7.30	5.73	6.34
**EB170**	4.76	8.34	7.82	6.16	7.74
**EB171**	4.77	7.94	7.38	5.98	7.20
**EB173**	5.12	9.35	8.75	6.69	8.51

Table S3. The correlation matrix for theoretically obtained lipophilicity parameters of tested compounds

	iLOGP	XLOGP3	WLOGP	MLOGP	SILCOS-IT
iLOGP	1.000	0.680	0.703	0.832	0.917
XLOGP3		1.000	0.998	0.972	0.900
WLOGP			1.000	0.979	0.917
MLOGP				1.000	0.972
SILCOS-IT					1.000

Table S4. The correlation between the molecular descriptors and the R_{M0} values for betulonic acid and its amide derivatives

Compound	Molecular descriptors	Equation	*r*
**betulonic acid**	*MW*	R_{M0} = 225.21 MW^2-2471.9MW+7248.3	0.963
	RefM	R_{M0} = 76.789 MW^2-843.42MW+2456.2	0.949
**EB170**	*HA*	HA = -19706 R_{M0}^2+21.73 R_{M0}-57.271	0.684
**EB171**	*HD*	HD = 1.6764 R_{M0}^2-19.04 R_{M0}+54.489	0.612
**EB173**	*RB*	RB = 2.1355 R_{M0}^2-22.512 R_{M0}+61.953	0.783
	TPSA	$TPSA$ = -1.4233 R_{M0}^2+ 10.82R_{M0}+30.32	0.367
	Caco-2 perm	$Caco\text{-}2\ perm$ = -0.1189 R_{M0}^2+1.2964 R_{M0}-2.2163	0.774
	Kp	Kp = -0.0423 R_{M0}^2+0.4754 R_{M0}-4.0832	0.345
	BBB	BBB = -1.1491 R_{M0}^2+12.681 R_{M0}-35.331	0.746
	PS	PS = -1.8637 R_{M0}^2+20.455 R_{M0}-57.456	0.892

Table S5. The correlation between the anticancer activity (IC_{50}) and the R_{M0} values for betulonic acid and its derivatives

Compound	Cell line	Equation	*r*
**betulonic acid**	C32	IC_{50} = 190.28 R_{M0}+976.03	0.962
**EB171**	Colo829	IC_{50} = 129.51 R_{M0}+686.05	0.858
**EB173**	A375	IC_{50} = 188.28 R_{M0}+1029.1	0.848
	MCF-7	IC_{50} = 413,11 R_{M0}+2242.6	0.792
